# Gate induced quantum wires in GaAs/AlGaAs heterostructures by cleaved edge deposition

**DOI:** 10.1038/s41598-021-01130-8

**Published:** 2021-11-05

**Authors:** L. Alt, C. Reichl, M. Berl, W. Dietsche, W. Wegscheider

**Affiliations:** Laboratory for Solid State Physics, ETH Zürich, 8093 Zürich, Switzerland

**Keywords:** Nanowires, Electronic and spintronic devices

## Abstract

Electric conductors with dimensions reduced to the nanometer scale are the prerequisite of the quantum devices upon which the future advanced electronics is expected to be based. In the past, the fabrication of one-dimensional (1D) wires has been a particular challenge because they have to be defect-free over their whole length, which can be several tens µm. Excellent 1D wires have been produced by cleaving semiconductors (GaAs, AlGaAs) in ultra high vacuum and overgrowing the pristine edge surface by molecular beam epitaxy (MBE)^1,2^. Unfortunately, this cleaved edge overgrowth (CEO) technique did not find wide-spread use because it requires a series of elaborate steps that are difficult to accomplish. In this Letter, we present a greatly simplified variation of this technique where the cleaving takes place in ambient air and the MBE overgrowth is replaced by a standard deposition process. Wires produced by this cleaved edge deposition (CED) technique have properties that are as least as good as the traditional CEO ones. Due to its simplicity, the CED technique offers a generally accessible way to produce 1D devices.

## Introduction

The molecular beam epitaxial (MBE) growth and the atomically flat cleave make the CEO technique ideal for confining carriers to a 1D wire with atomic precision. In contrast to other 1D wire methods, multiple wires can be precisely aligned on-top of each-other, enabling interesting tunneling experiments, ideal to study electron-electron interactions and gain further insights into the Luttinger-Liquid theory^3,4^. A substantial mean free path can be reached with high sample quality, due to the optimized MBE growth, ensuring ballistic transport. Additionally, this method has the advantage of the wire being coupled to two-dimensional electron gas (2DEG) reservoirs, ensuring good contacts. Further attention to the technique comes with the recent interest in combining a 1D wire and a superconductor. Adding a superconductor onto the cleaved edge has been predicted to create a topological superconducting state. The basic setup needed is an 1D wire with strong spin-orbit coupling, a s-wave superconductor deposited on the cleaved edge and a magnetic field to break time-reversal symmetry^5^. Near the ends of the wire at the interface between the topological superconducting state and the trivial state, Majorana Fermions are predicted^6^. The high interest present in this field requires the need for a reliable yield of high-quality quantum wires.

The concept of CEO might seem relatively straightforward, but this simplicity is misleading since the CEO method brings various experimental challenges. The most significant being the 2nd MBE overgrowth, where the sample has to be cleaved with atomic flatness, within the MBE apparatus and then immediately overgrown with a high purity (110) growth to ensure a defect-free lateral confinement. The need for a cleaving apparatus within a high purity MBE system and having to thin down the samples to ensure atomically flat cleaves are just two of many challenges in the CEO process^7^. In our work with CEO we have identified the facet, which forms at the edge of the sample where the overgrowth is not well-defined, as the most serious issue, possibly leading to an amphoteric character of doping. This difficulties explain the very limited amount of CEO samples circulating. Thus, an approach simplifying the device fabrication process and enhancing the reliability of the CEO samples is highly desirable.

We found that by cleaving the sample outside the MBE system and depositing a side-gate onto the (110) cleaved edge, we can induce a ballistic quantum wire with a high yield, eliminating the problems of the 2nd MBE overgrowth. The confinement potential in this novel method is induced by applying a positive side-gate voltage, whereas in the traditional CEO method the potential gets created by a doping layer in the (110) growth. By simplifying the CEO method and overcoming the most significant part of the experimental challenge, the accessibility of such high-quality one-dimensional quantum wires is no longer restricted to researchers operating highly specialized MBE systems. To distinguish the novel method, we call it gate-induced cleaved edge deposition (CED). With this new method ballistic transport features with no deviation from the conductance quantization are achieved, in contrast to most traditional CEO samples^8^.

**Figure 1:**
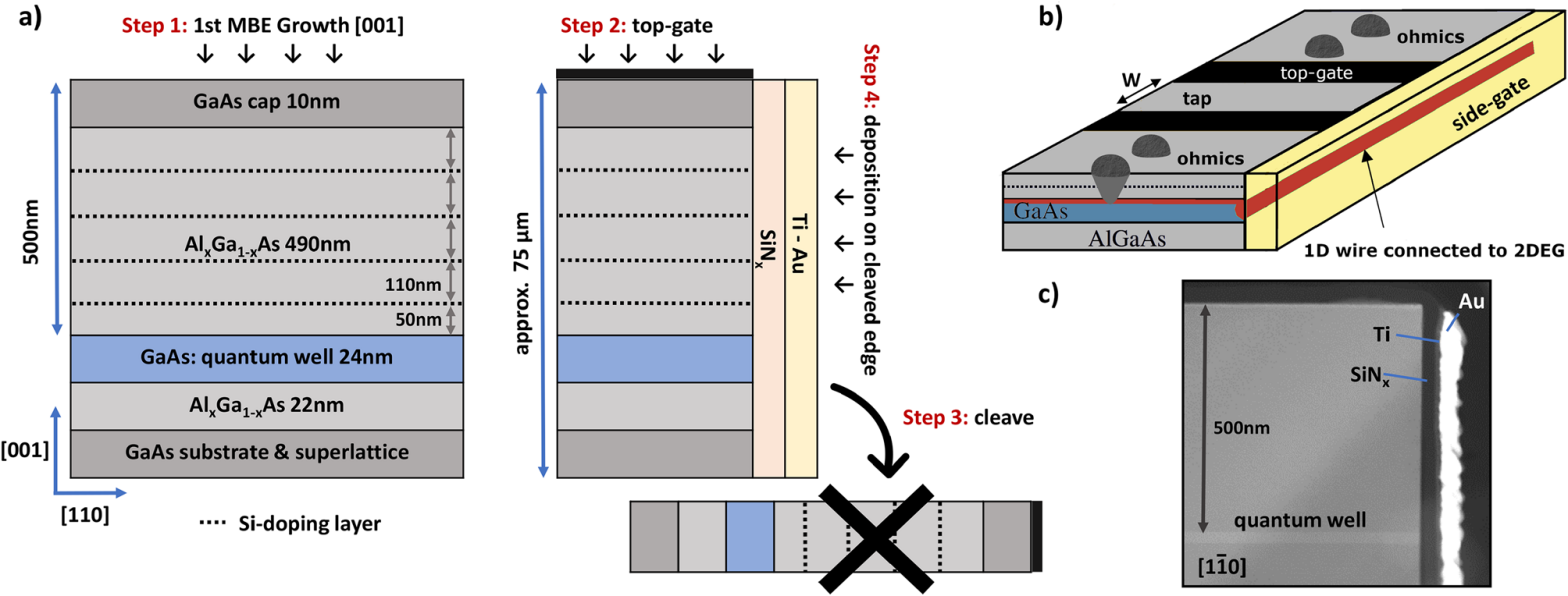
(**a**) Stepwise illustration of the novel cleaved edge deposition method. Step one consists of the first MBE growth along [001] direction resulting in a high mobility 2DEG buried 500 nm under the surface. In the second step top-gates are defined on the (001) surface and the substrate is thinned down to approximately 75 μm. In steps 3 and 4 the experimentally challenging 2nd overgrowth has been replaced by an ex-situ cleave and a side-gate deposition. (**b**) The 3D schematic illustrates the one-dimensional wire forming along the edge (highlighted in red) by applying positive side-gate voltages. By applying negative top-gate voltages the 2DEG underneath the top-gates can be depleted and the chemical potential in the 1D wires tuned. The wires are contacted over the 2DEG reservoirs to which they are coupled over the entire edge. (**c**) A HAADF TEM image of the side-gate deposition onto the cleaved edge.

## Fabrication

In the first step of fabrication, an Al_0.31_Ga_0.69_As/GaAs heterostructure is grown in [001] direction by MBE-growth, consisting of a 24 nm GaAs quantum well buried under 490 nm of Al_0.31_Ga_0.69_As and a 10 nm GaAs cap. Solely for the purpose of enhanced contactability, multiple silicon (Si) delta-doping layers are used to provide charge carriers. The high mobility 2DEG forming at this single-sidedly doped quantum well has a charge carrier density *N* = 1.77 × 10^11^ electrons/cm^2^ and a electron mobility μ = 4.93 × 10^6^ cm^2^/Vs at 1.2 K. In the second step, tungsten top-gates are evaporated and structured via e-beam deposition, photolithography and plasma etching. A lapping process is used to thin down the substrate to approximately 75 μm, which is highly beneficial to obtain atomically flat cleaves later on. Until here, the fabrication process is the same as for traditional CEO-samples, which will be overgrown within the MBE and is illustrated in the first two steps of Fig. 1a. The major difference are step 3 and 4, where the in-situ cleave and 2nd MBE overgrowth are replaced by less challenging steps. In contrast to the CED technique, steps 3 and 4 consist of cleaving under atmosphere, where a native oxide is inevitably formed, and depositing a side-gate onto the atomically flat cleaved edge. The side-gate consists of a 30 nm thick silicon nitride (SiN_*x*_) plasma enhanced chemical vapor deposition grown layer and a 10 nm/100 nm Ti/Au (e-beam evaporated) layers, deposited onto the cleaved edge under the angle of 70°. This prevents depositions onto the top-gates, which could lead to unwanted shorts. The schematic in Fig. 1b) illustrates the one-dimensional wire forming along the edge (highlighted in red), as a positive side-gate voltage is applied. Figure 1c) shows a high-angle annular dark-field transmission electron microscopy (HAADF TEM) image of the side-gate deposition onto the cleaved edge.

## Results and discussion

**Figure 2:**
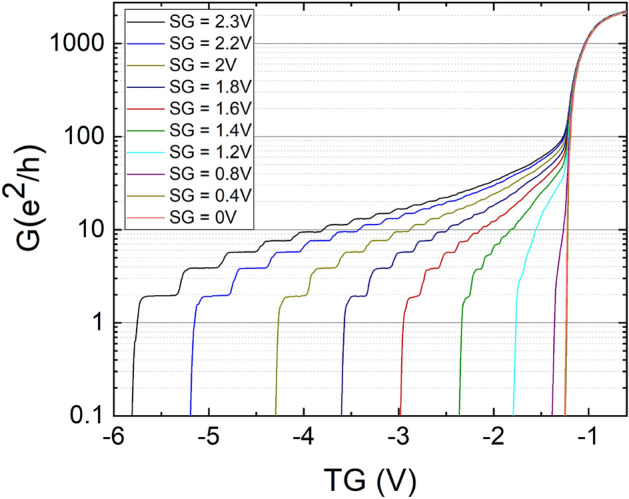
Dependence of conductance on the top-gate (TG) voltage for different side-gate (SG) voltages. At higher side-gate voltages 1D transport behavior on a cleaved edge deposition sample over a 2 μm wide wire at 25 mK with an excitation of 6 μV can be induced.

For CEO samples the lateral confinement is created by the doped overgrowth of the cleaved edge. In contrast to that for the CED samples the confinement is induced by applying a positive side-gate voltage. Figure 2 shows the effect of different side-gate voltages on the conductance plateaus of a CED sample. The quantization steps in the plot start to form with increasing positive side-gate voltage, getting broader and more pronounced. Remarkably, almost no deviation of the conductance (*G*) from the universal conductance quantization (*G*_0_) can be seen down to temperatures of 25 mK. This is in contrast to most other CEO samples studied, where the deviation is present but the origin is not clear and different explanations are given^8^.

**Figure 3:**
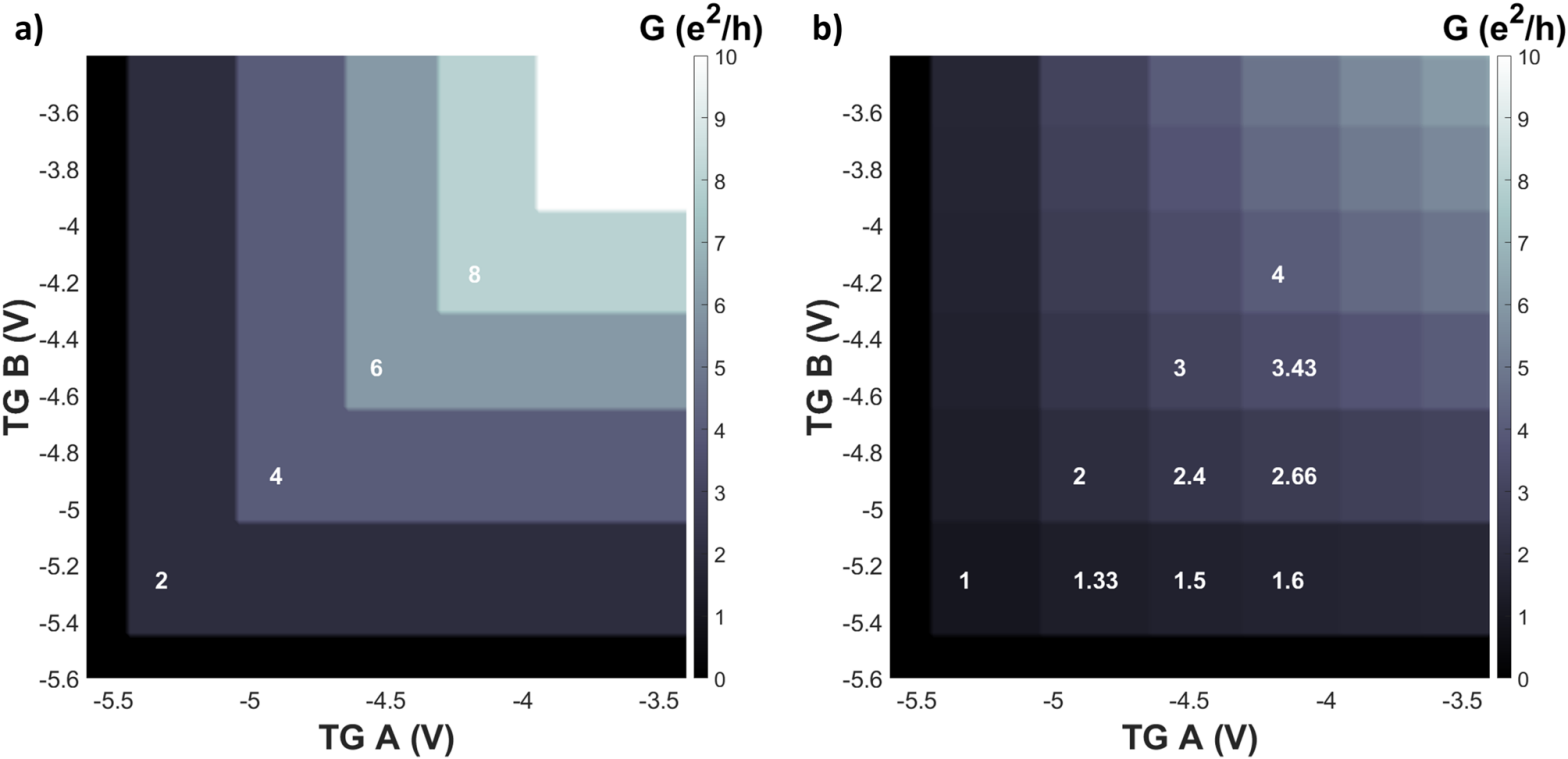
Calculated model of the series resistance of two wires A and B separated by a 2DEG tap, showing a contour plot of conductivity as function of both top-gate voltages. (**a**) Non-invasive tap: no scattering inbetween the wires leading to a ballistic behavior across both wires. (**b**) Invasive tap: the two wires A and B act like two resistances in series where each wire resistance is given by the parallel resistances of its occupied modes.

A method developed by De Picciotto et al. can be used to determine whether the transport is ballistic or diffusive^9^. For that two wires are coupled in series separated by a 2DEG section of width *W*, called tap, as shown if Fig. 1b). Using the principle, we studied different 2DEG tap widths between two top-gates (A and B). The gates A and B are of 2 μm width and tune the number of modes in wires A and B, respectively. They are separated by 2DEG taps where *W* = 3.1 μm, 7.7 μm and 27.2 μm. If electrons couple from the source 2DEG into 1D wire modes, pass wire A, the tap and wire B without back-scattering or interacting 2DEG modes of the tap (non-invasive), ballistic transport occurs. In this case the two wires in series behave like one long wire. Figure 3 a) depicts a contour plot of conductivity as a function of both top-gates A and B, based on a resistance model of ballistic transport. The top-gate values indicating the onset of a new sub-band have been taken from our measurements. In the ballistic case, with no scattering, the wire with the lower number of modes will define the conductance over both wires. The modeled conductance is thus given by $G = min \left( G_A, G_B\right)$. The modes carrying the current through both wires and the tap, are regarded as in parallel to each other.

If the 2DEG tap between the two wires is invasive, meaning the electrons scatter into 2DEG modes after passing wire A, the system is in diffusive transport regime. The plot of Fig. 3b) depicts a contour plot based on the resistance model for diffusive transport, showing a typical check-board pattern. Here due to the invasiveness of the tap, the two wires, A and B, act like two resistors in series, with each wire’s resistance is given by the parallel resistances of its modes.

**Figure 4:**
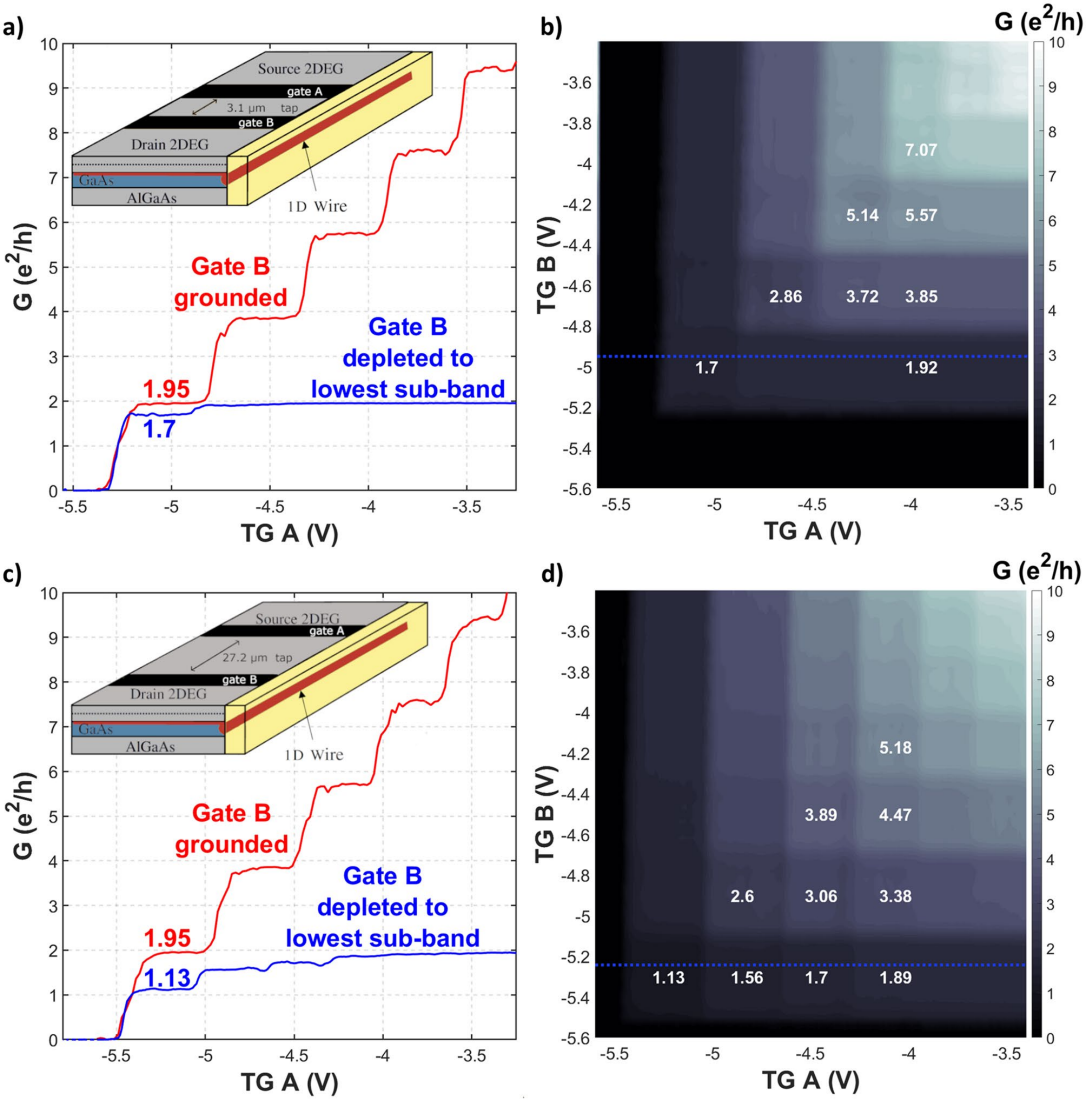
Measurements at 25 mK using an ac excitation of 6 μV over two wires A and B separated by a tap of two different widths. (**a**) Conductance dependence of top-gate A at a side-gate voltage of 2.3 V, for solely wire A (top-gate B on ground, red line) and for wire B tuned to the lowest sub-band with *W* = 3.1 μm (blue). A mixture of ballistic and diffusive transport can be observed. The inset shows a schematic of the device. (**b**) Contour plot of conductivity as function of both top-gate voltages. The blue line shows the cut from a) where wire B is tuned to the lowest sub-band. (**c**, **d**): Analog to (**a**) and (**b**) but with *W* = 27.2 μm. A pronounced check-board pattern indicating mostly diffusive transport can be observed.

Figure 4 shows the results of our measurements at 25 mK for two different tap lengths and the side-gate fixed at a voltage of 2.3 V. For the plots a) and b) the tap width *W* is 3.1 μm. A mixture of ballistic and diffusive transport can be observed. The conductance value, which can be read out from the linecut, is close to 2 *e*^2^/h, as expected from the model for ballistic transport. Still, check-board patterns are visible, indicating a contribution of a diffusive component. In Fig. 4c and d *W* is 27.2 μm and a pronounced check-board pattern is visible, clearly indicating diffusive transport. Also, the conductance value drops to almost *e*^2^/h as expected from two diffusive wires in series. The conductance values are averaged by fitting a plane into each square of the respective check-board and are quantified in white.

For the sake of simplicity, we will study the case of equal wire conductances ($G_A = G_B = n\times \frac{2e^2}{h}$). In order to analyze the ratio between the ballistic and diffusive component in transport we can use the Landauer formula $G_n= \frac{e^{2}}{h} \, n \, \left( 1+T_n \right)$, where $T_n$ is the transmission probability of *n* modes^10^. From the conductance value $G_n$, measured over both wires A & B in the same mode *n*, one can calculate the transmission probability of said mode through the tap. As a reference, the conduction values $\text {G}_0$ of wire A without wire B are used, depicted in red in Fig. 4a,c. The Landauer formula can now be written as $G_n= \frac{\text {G}_0}{2} \, n \, \left( 1+T_n \right)$ and the transmission probability can be calculated from the measured conductance values through wire A & B with respect to the values of only wire A. For purely ballistic transport, the transmission probability would be 1 for each mode. In contrast, diffusive transport resulting in the ohmic addition of resistances of the two wires, has a transmission probability of 0. The calculated transmission probabilities of the first four modes with *W* = 3.1, 7.7, and 27.2 μm are summarized in table 1. From these results one can see a clear evolution from ballistic to diffusive dominated transport as the tap size increases.

**Table 1:** Transmission probability of the first four modes, calculated with the Landauer formula, from the measured conductance trough wire A and B tuned to the same mode, separated by taps of various widths: (**a**) *W* = 3.1 μm (**b**) *W* = 7.7 μm (**c**) *W* = 27.2 μm.

# Mode(*n*)	$n \, G_{0} \left( e^{2}/h\right)$	$G_{n} \left( e^{2}/h\right)$	$T_{n} \left( \mathrm {calc}\right)$
(a) Tap of 3.1 μm between wire A & B			
1	1.95	1.7	0.74
2	3.85	2.86	0.49
3	5.75	5.14	0.79
4	7.6	7.07	0.86

# Mode(*n*)	$n \, G_{0} \left( e^{2}/h\right)$	$G_{n} \left( e^{2}/h\right)$	$T_{n} \left( \mathrm {calc}\right)$
(b) Tap of 7.7 μm between wire A & B			
1	1.93	1.45	0.5
2	3.84	3.1	0.61
3	5.76	4.49	0.55
4	7.6	6.2	0.63

# Mode(*n*)	$n \, G_{0} \left( e^{2}/h\right)$	$G_{n} \left( e^{2}/h\right)$	$T_{n} \left( \mathrm {calc}\right)$
(c) Tap of 27.2 μm between wire A & B			
1	1.95	1.13	0.16
2	3.81	2.6	0.36
3	5.67	3.89	0.37
4	7.55	5.18	0.37

**Figure 5:**
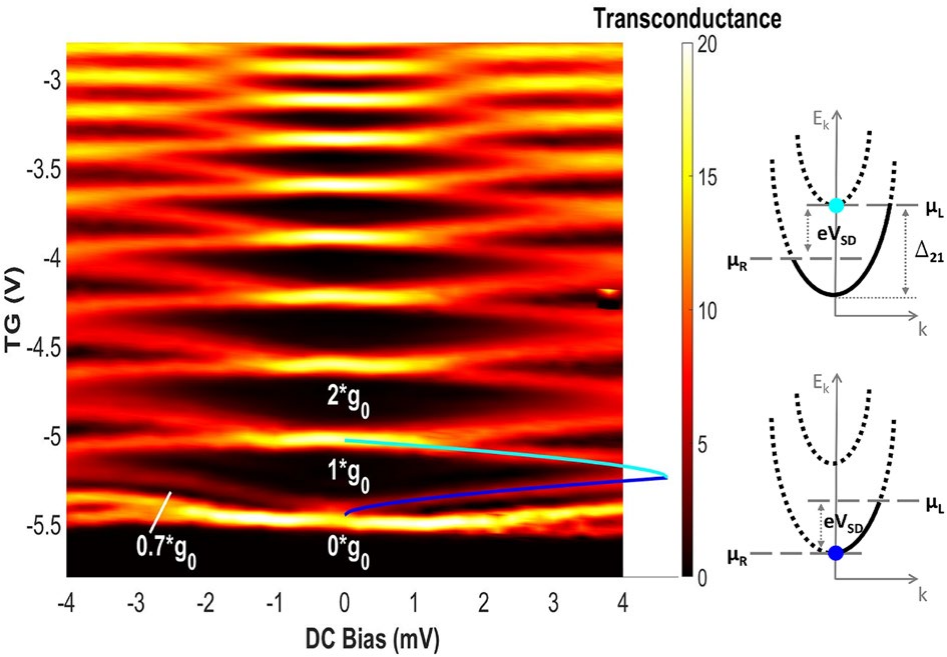
Transconductance dependence on dc bias and top-gate voltage of a 2 μm wide wire at 25 mK with 6 μV ac excitation. Transconductance is plotted as a function of the dc bias and top-gate voltage giving rise to diamond shaped, dark areas separated by regions of high transconductance. Using the model of Picciotto et al.^13^ the shape of the transitions is calculated and is depicted by blue and magenta lines. Along the transconductance transitions either the chemical potential of the source $\mu _L$ or the drain $\mu _R$ is aligned with a sub-band, as shown in the schematic of the parabolic energy dispersion.

In addition, we have performed dc bias measurements on a single CED wire. Figure 5 shows the transconductance ($dG/dV_{TG}$) plotted as a function of the additional dc bias between source and drain ($V_{SD}$), and the top-gate voltage ($V_{TG}$). The transconductance describes the sensitivity of the conductance with respect to the charge density in the wire. The dark areas in the plot characterize regions of different conductance values, which are separated by high transconductance regions (red-white) where the gate voltage dependence is strong. The different conductance values are given in multiples of the conductance quantum, $\text {G}_0 = 2 \, e^{2}/h$. Between the zero-conductance state and the first step, a clear conductance peculiarity, called ,, 0.7 structure”, can be seen at $\sim 0.7 \, \text {G}_0$, which becomes more pronounced at larger applied biases. This bias dependence has already been reported in quantum point contacts (QPC’s)^11^, and in similar dc bias measurements on CEO wires^12,13^. In contrast to a QPC where the plateaus in the differential conductance appear as diamonds of zero transconductance centered around zero bias voltage, the plateaus in wires form the observed curved diamonds with edges distorted towards lower top-gate voltages. Rössler et al. have shown such a change in shape of the differential conductance diamonds for two QPCs’s, one of them having a longer quantum wire-like geometry^14^. This distortion has been modeled by De Picciotto et al. taking into account the requirement of charge neutrality, as the charge density in the wire is nearly independent of the dc bias^13,15^. In their model, the 1D spin-degenerate density of states of the wire, resulting from a parabolic energy dispersion, is capacitively coupled to the top-gate. As a source-drain bias is applied, the chemical potential $\mu _L$ of the source (left reservoir) is changed in respect to the drain (right reservoir, $\mu _R$) and states with positive wave vector $k_x$ travel from source to drain with the electrochemical potential $\mu _L$ and vice versa. Along a transition region (high transconductance) either the number of electrons moving form source to drain or vice versa is fixed, making it possible using this constraint to calculate the shape of the transition regions with their model. With the sub-band spacing of $\Delta _{21} = 4.64 \, \mathrm {meV}$ and the gate voltage range of $\Delta V_{tg} = 0.3 \, \mathrm {V}$ extracted from our transconductance plot, we computed the magenta and blue curve and calculate a geometrical capacitance of $C_G = 35.6 \, \mathrm {aF}$. The values for the sub-band spacing are taken from the data where the curves intersect at non-zero bias voltages, and the conductance transitions meet. At this point, the chemical potential of the source is aligned energetically to one sub-band, and the other neighboring sub-band is aligned with the drain. Along the transition described by the magenta curve, $\mu _L$ is aligned with the second sub-band and the amount of electrons moving from source to drain is fixed. For the transition along the blue curve $\mu _R$ is aligned with the first sub-band and there are only electrons moving from source to drain, while no electron flow in the opposite direction (fixed). In figure 5 the energy dispersion for the first two sub-bands is illustrated pointing out the alignments of the chemical potentials for both conductance transitions described by the magenta and blue line. These computed curves fits our data well and, thus, coincide with the expected behavior of a one-dimensional wire.

Similarly to De Picciotto’s results^13^, the “0.7 structure” in our non-linear transport measurements is adherent to the transition described by the blue line, where the current is unidirectional. Different explanations have been proposed to explain the origin of the “0.7 structure” in QPC’s and wires. Spontaneous spin polarization associated with the enhanced role of e-e interaction in 1D-transport^11^, ferromagnetic spin coupling^16^ and for wires De Picciotto et al. suggest from their study a possible link to the screening properties of a quantum wire, in which all electrons are unidirectional^13^. However since the “0.7 structure” is also observed at elevated temperatures in linear response measurements where the unidirectionality is unlikely, it’s origin remains not fully explained^12^.

It seems surprising for the CED technique to create such excellent 1D wires. Due to the cleaving under atmosphere a native oxide layer is inevitable. For the GaAs (110) surface it has been shown that even a monolayer of oxygen can lead to a fermi-level pinning, similar to the Schottky barrier pinning created by metal deposition^17^. We put forward the hypothesis, that due to this barrier, the wire gets pushed away from the interface, and by doing so, scattering with surface roughness at the oxide interface is suppressed.

## Conclusion and outlook

We developed a new, simplified technology to create 1D quantum wires in high quality GaAs heterostructures. This technology overcomes the experimental challenges and the related problems resulting from the second overgrowth in the traditional CEO technique. The CED technology is more widely accessible since the only prerequisite is a high purity 2DEG.

In this work, we show the proof of concept for this method. Using a side-gate deposited onto a cleaved edge, we could induce a confinement potential leading to a 1D wire showing quantization steps. The conductance deviation, often seen in quantum wires, is much less pronounced in our samples and is only noticeable in higher modes. Using two gates in series we could show that for different 2DEG tap lengths between the gates, the transport behavior changes from ballistic to the diffusive regime with increasing tap length.

Using the CED technique with a combination of both front- and back-gates would allow for interesting novel quantum wire devices^18^. Among those are coupled ballistic wires, which can be contacted and gated independently. Additionally, this technology can be easily transferred to other material systems, e.g. indium arsenide wires, which would be highly beneficial for the study of Majorana-physics due to the strong spin-orbit interactions. Also the CED technology is scalable which allows to create 1D wire networks.
